# A Particle Model for Prediction of Cement Infiltration of Cancellous Bone in Osteoporotic Bone Augmentation

**DOI:** 10.1371/journal.pone.0067958

**Published:** 2013-06-26

**Authors:** Ehsan Basafa, Ryan J. Murphy, Michael D. Kutzer, Yoshito Otake, Mehran Armand

**Affiliations:** 1 Department of Mechanical Engineering, Johns Hopkins University, Baltimore, Maryland, United States of America; 2 Laboratory for Computational Sensing & Robotics, Johns Hopkins University, Baltimore, Maryland, United States of America; 3 Johns Hopkins University Applied Physics Laboratory, Laurel, Maryland, United States of America; 4 Department of Orthopaedic Surgery, Johns Hopkins University, Baltimore, Maryland, United States of America; University of California, San Diego, United States of America

## Abstract

Femoroplasty is a potential preventive treatment for osteoporotic hip fractures. It involves augmenting mechanical properties of the femur by injecting Polymethylmethacrylate (PMMA) bone cement. To reduce the risks involved and maximize the outcome, however, the procedure needs to be carefully planned and executed. An important part of the planning system is predicting infiltration of cement into the porous medium of cancellous bone. We used the method of Smoothed Particle Hydrodynamics (SPH) to model the flow of PMMA inside porous media. We modified the standard formulation of SPH to incorporate the extreme viscosities associated with bone cement. Darcy creeping flow of fluids through isotropic porous media was simulated and the results were compared with those reported in the literature. Further validation involved injecting PMMA cement inside porous foam blocks — osteoporotic cancellous bone surrogates — and simulating the injections using our proposed SPH model. Millimeter accuracy was obtained in comparing the simulated and actual cement shapes. Also, strong correlations were found between the simulated and the experimental data of spreading distance (R^2^ = 0.86) and normalized pressure (R^2^ = 0.90). Results suggest that the proposed model is suitable for use in an osteoporotic femoral augmentation planning framework.

## Introduction

Osteoporotic hip fractures are responsible for billions of dollars of annual hospitalization and treatment costs in the United States [1], [2]. Current preventive measures include prescription of a variety of drugs, use of hip protectors and special exercises for strengthening muscles [3]. Most of these techniques have a limited effect due to their costs and/or lengthy treatment requirements. A potential near-term preventive measure for osteoporotic hip fractures is femoroplasty — augmentation of the mechanical properties of the proximal femur using bone cement [2]–[4]. Although promising, this method requires precise planning and execution and is not yet part of clinical practice. Introducing a large volume of cement into the bone can result in necrosis, i.e. tissue death due to lack of blood supply, as the polymerization process of the bone cement is highly exothermic [3], [5]. On the other hand, small volume injections can be of no effect if not planned accurately [6]. As a result, augmentation of the bone should be performed by employing a limited amount of strategically placed cement. A successful planning framework should hence include a module for predicting cement diffusion inside porous cancellous (spongy) bone in order to assess the outcome of hypothetical augmentations.

Modeling of rheological properties and flow of bone cement has been the topic of research for the past few years. Among the approaches are finite element [7], [8], analytical [9] and heuristic models [10]. In the more recent years, particle models have gained popularity for modeling fluid flows. These models provide a Lagrangian view of the flow, where the simulation (observer) tracks the motion of fluid particles, as opposed to tracking the change of variables inside fixed grid cells in space as in the Eulerian view. Among these models' advantages over grid-based methods are the inherent conservation of mass, no need for creating and maintaining a grid structure and fast computations of equations of motion. Because of their superior simulation speeds, particle models are of utmost interest in the graphics community and they have been used to model fluids and flow of colloids such as sand [11]–[14]. Heuristic approaches are taken in these methods to model the particle-particle and particle-environment interactions that best serve the specific application of interest.

An alternative to the empirical particle models is the method of Smoothed Particle Hydrodynamics (SPH), which is a way of approximating continuum field quantities by several discrete particles. First introduced by Lucy [15] and Gingold and Monaghan [16], SPH saw its first application in modeling astrophysical phenomena. Several other SPH applications have been realized since, including modeling of fluid flows such as free surface water simulation [17], [18], melting and viscoplastic objects [19], [20] and porous flow realization [21]–[24]. In most of the aforementioned works visual aesthetics take precedence over physical realism of the flow and there is usually no quantitative validation using experimental data.

As we are interested in predicting the dispersion of viscous cement inside cancellous bone, we used SPH for porous flow modeling. Of particular importance is the capability of the model to predict the end shape that the cement assumes after a certain amount is injected inside a porous medium. We used an implicit numerical integration method to cope with the stability problems arising when modeling highly viscous materials. Hence, in incorporating the Navier-Stokes equations into the SPH formulation, we modified the standard viscosity model to make the formulation computationally more efficient. We tested the model's simulation capabilities in two scenarios: Darcy flow simulation and modeling of bone cement injection in porous media. For the latter scenario, simulation results were compared with experimental data of injection of Polymethylmethacrylate (PMMA) bone cement inside surrogate porous bone models. The following sections describe the formulation and the validation simulations followed by the obtained results.

## Methods

### Particle Fluid Model

SPH is a way of interpolating field quantities in discrete particle systems. For these systems, continuous quantities such as velocity or density field are assumed to be known at some discrete locations (particles) and one can approximate their values in any other given point in space by employing the so-called ″smoothing kernel″ function W, as in Eq. 1 [16].
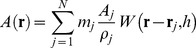
(1)


Here A is the field quantity, m is particle mass, ρ is density, N is the total number of particles, **r** is the position in space, and h is the smoothing length. The smoothing kernel function W distributes, in a (small) neighborhood, the field quantities around particles. It is shown that if the function is even, i.e. W(x) = W(−x) for any x position in space, as well as normalized, i.e. has unit integral over its entire domain, then Eq. 1 approximates the field quantities with second order accuracy [17]. Although the summation in Eq. 1 should be evaluated for all the particles, the kernel functions are usually designed to have finite support to reduce the computational load. It is easy to generalize Eq. 1 for evaluating derivatives of field quantities as well. In most SPH simulations masses of particles are constant, leading to inherent conservation of mass, so one can evaluate each particle's density using Eq. 2.
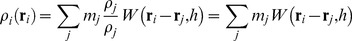
(2)


To use the SPH formulation for modeling of fluid flow, the Navier-Stokes equations [25] can be used. The equations for a viscous, incompressible fluid can be written as Eq. 3.
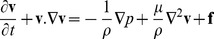
(3)


In Eq. 3, **v** is the velocity field, p is pressure, μ is viscosity, and **f** is the external body force (force per unit mass, or acceleration). The left hand side of Eq. 3 can be simplified as in Eq. 4,
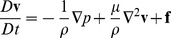
(4) and because of the Lagrangian nature of the particle models, i.e. the assumption that the particles move locally with the fluid flow, the term D**v**/Dt can be directly used as the time rate of change of the velocity of particles [17]. Once all the right hand side terms are evaluated for each particle, its position and velocity can be updated for the next time step in the numerical simulations.

Evaluating the pressure gradient using the standard SPH formula of Eq. 1 for derivatives results in non-symmetric pressure gradients [26]. Therefore, we used the symmetric pressure gradient (Eq. 5) proposed by Monaghan [26].
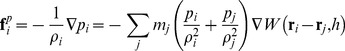
(5)


Here **f** ^p^ is the pressure gradient force vector. Before Eq. 5 can be evaluated for each particle, pressure values at each particle location must be known. This can be achieved by using an equation of state, in the form of Eq. 6 [23], [27].
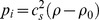
(6)


In Eq. 6, c_s_ is the speed of sound in the fluid and ρ_0_ is the density at rest of the fluid. Using the actual speed of sound in the fluids results in very stiff equations that limit the integration time step size prohibitively. Therefore, much smaller values are typically used so that volume is preserved within an acceptable range and computational speed is not compromised [28]. We used a value of 1 m/s for c_s_ — about two orders of magnitude larger than the usual fluid flow velocities that occur during cement injection.

To account for the energy loss due to viscosity, the approximation in Eq. 7 is usually employed [29], [30].
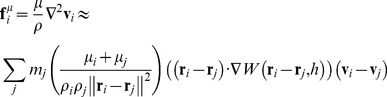
(7)


In the above equation, **f** ^μ^ is the viscosity force vector. However, as will be explained shortly, we modified Eq. 7 to model highly viscous fluids such as bone cement. If one employs a typical explicit numerical integration method, the stability criteria dictate prohibitively small time step sizes for highly viscous fluids [28], [31], [32]. Working viscosity of bone cement ranges from 100 to 1000 Pa.s on average [10], [33], which is several orders of magnitude larger than the viscosity of water (0.001–0.01 Pa.s) that is usually used in its SPH simulations [22], [23]. Therefore we used the implicit central difference method for integration, summarized in Eq. 8:
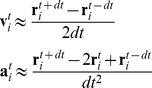
(8) Where t-dt, t, and t+dt represent the previous, current, and future states and **a**_i_ is the acceleration (left hand side of Eq. 4). This way, the future position of each particle can be found as a function of its previous and current positions. Since the forces acting on the particles are functions of velocity as well as position, this is an implicit problem involving the solution to an equation of the form M**r** ^t+dt^ = **b** where M is a sparse 3N-by-3N matrix, **b** is a 3N-by-1 vector, and **r** ^t+dt^ is the 3N-by-1 vector of positions at time t+dt of all the particles. M is sparse because motion of each particle is affected by a small number of neighboring particles. In general, solutions to these types of problems are computationally expensive. In the special case of velocity-dependent forces acting on each particle being functions of its own velocity only, matrix M becomes diagonal and the solution can be computed fast. Eq. 9 was used to satisfy this criterion,
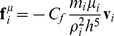
(9) where the scaling factor C_f_ can be adjusted to achieve the desired flow behavior. The viscous force can be interpreted as a force opposing the motion of particles, proportional to their velocity and viscosity. This approximation seems to yield reasonably accurate results for modeling of cement flow inside porous media.

The porous medium was modeled by fixing some of the fluid particles to represent the solid regions [22], [28], [29]. These fixed particles were treated as fluid particles for calculating moving particles' density and pressure, but their density and pressure as well as position and velocity ( = zero) were kept fixed over time.

The choice of the kernel function is crucial to SPH simulations. We chose the cubic spline kernel for our density calculations [34]. Here we have W(||**r**−**r**_j_||,h) = w_1_(||**r**−**r**_j_||/h) with w_1_(q) being a kernel with support radius of 2 h and given by Eq. 10:
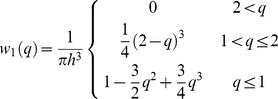
(10)


The gradient of the cubic spline kernel vanishes at the origin which results in particles clumping together, if it is used for pressure gradient calculations [17]. Therefore, for pressure calculations, we chose a kernel with non-vanishing gradient as in Eq. 11 [35].
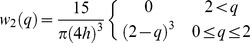
(11)


### Darcy Flow Simulation

To test the validity of the model, we simulated one-dimensional flow of viscous fluids inside isotropic (i.e. with direction-independent properties), randomly porous media and used Darcy's formula to calculate their permeability. Darcy's constitutive equation describes the flow of a viscous fluid through a porous environment. For a steady state, one dimensional flow under constant body force, Darcy's formula reduces to Eq. 12 [29]:
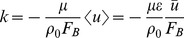
(12)


**Figure 1:**
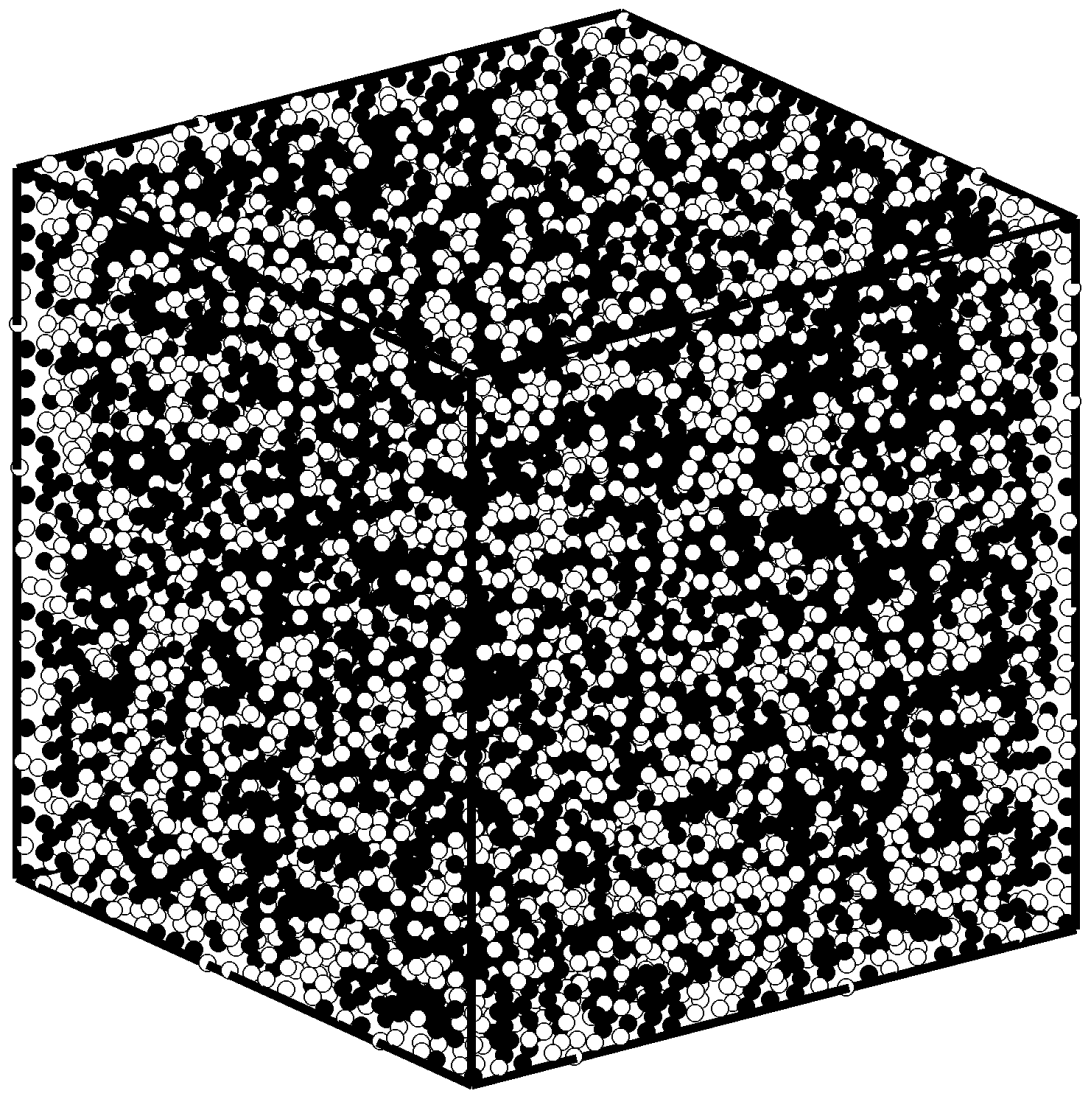
Initial particle arrangement for Darcy flow simulation. Black particles represent the porous medium (porosity of 0.5) and white particles represent the fluid.

Here, k is permeability, F_B_ is the applied body force, <u> is the Darcy velocity, ū is the average flow velocity, and ε is porosity. To simulate such a flow, particles were initialized in a rectangular grid of the size 25X25X25 particles (Figure 1). A number of particles were randomly fixed to the grid, representing the solid regions. Porosity was determined by 1-(#fixed particles/#all particles) and was varied between 0.5 to 0.9, in 0.1 steps. Particles were assumed to occupy a volume of d^3^, with d being the initial particle spacing, and therefore were given a mass of ρ_0_d^3^ with ρ_0_ = 1.18 gr/cm^3^. Two initial particle spacings of 0.5 and 1 mm and two viscosity values of 10 and 100 Pa.s were simulated. For each fluid, particles were initialized and a constant body force was applied to the system. The kernel radius was set to h = d/2. Periodic boundary conditions were applied to the model: if in each simulation time step any particle escaped the domain from one side, it was removed from the list and an identical particle was added to the opposite side with the same velocity and density [29]. Sufficient time was given to the simulation for the flow to reach steady state. To avoid edge effects, the average velocity of particles passing through a plane perpendicular to the mid section of the domain was calculated. The ratio of ū/F_B_ was determined by the slope of the best-fit line between average velocity and body force. Permeability was then calculated using Eq. 12 and it was converted to dimensionless permeability by employing the procedure described by Jiang et al. [29].

### Foam Block Tests

#### Experiments

To further validate the model, we performed injection experiments in a setting similar to osteoporotic bone augmentations with PMMA bone cement, inspired by the experiments of Loeffel et al. [36]. The goal was to perform precisely controlled injections using our in-house cement injection device [37] and compare the results with the corresponding simulations. Eight porous foam blocks (Open Cell Blocks, Sawbones, Vashon, WA) were cut in blocks of approximately 65X65X40 mm^3^. Each block, representing cancellous bone, was tightly enclosed in a plexiglass shell of 5 mm thickness, acting as the cortical shell. On each side of the shells we drilled a 3-mm vent hole, temporarily blocked the holes, and filled the entire combination with melted dairy butter representing the bone marrow [36]. The vent holes provided an escape path for the butter when pressed out by the cement during the injection. Each block was also equipped with eight 2-mm steel balls as registration landmarks. CT scans (Aquilion 64, Toshiba Medical Systems, Japan) were then acquired from the blocks with 0.5 mm slice thickness and 0.47 mm in-plane resolution and the blocks were refrigerated to maintain solidity of the dairy butter.

**Figure 2:**
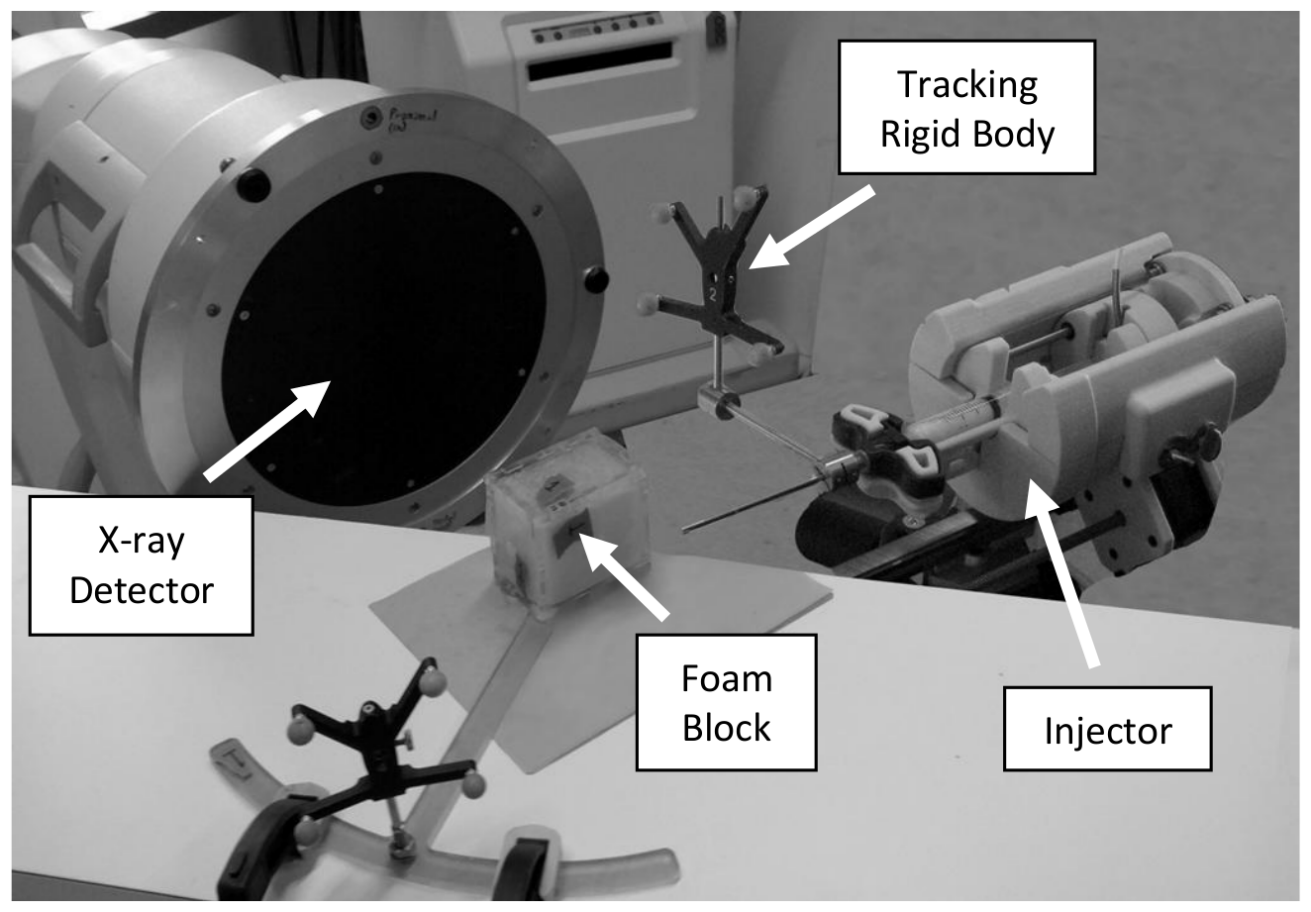
Experimental setup.

The day before each experiment, the blocks to be tested were removed from the refrigerator and left at room temperature for the butter to soften. At the time of experiments, we placed each block on a testing table in the field of view of an X-ray image intensifier (mobile C-arm (OEC9600, GE Healthcare, UK)) and opened the vent holes. To guide the injection device into the center of each block, we used an in-house navigation and tracking system [38]. To facilitate the navigation, each block was equipped with an extension that was used to mount a tracking rigid body (Figure 2). The registration was performed by first digitizing the steel balls and recording their coordinates in the tracking system frame. Then, knowing the corresponding coordinates in the CT volume frame, we found the transformation between the two coordinate systems. Using this resulting transformation, the navigation system identified the pre-determined center of the block as the target point of injection, in the tracking system coordinate frame. Spineplex radiopaque bone cement (Stryker Instruments, Mahwah, NJ) was prepared and a 5 ml syringe was filled with the cement. A 9-cm, 11G cannula (Stryker Instruments, Mahwah, NJ) was attached to the syringe and the combination was mounted on the automatic injection device. After an initial wait period of ten minutes, cement viscosity was estimated by pressing out the cement with 0.05 ml/s flow rate for 5 seconds and measuring the average syringe pressure in the last second (the plateau region). The wait time was determined based on our preliminary tests on the cement viscosity at a room temperature of 21PC (fixed for all the tests). Hagen-Poiseuille law (Eq. 13) was used to estimate the cement viscosity [36]. The formula relates the pressure drop between two points in a viscous fluid flowing inside a tube to the viscosity and flow rate of the fluid and the dimensions of the tube.
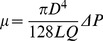
(13)


In Eq. 13, D is the cannula inner diameter, L is the length of the cannula, Q is the volumetric flow rate, and ΔP is the pressure drop between the two ends of the cannula. Four target viscosity levels of 50, 100, 200 and 400 Pa.s were tested. For the two lower viscosity levels, the estimation was done once and the injection time was determined by interpolating the values that were obtained in the preliminary tests. For the two higher viscosity levels, we performed an additional viscosity estimation, one minute after the first one, for better interpolation accuracy. At this point, we guided the needle, using a second tracking rigid body attached to it, to the center of the porous block and the control system started the injection at the time that the viscosity was expected to have reached the desired value. At the same time, the control software started the recording of X-ray video sequence of the C-arm as well as the syringe pressure. An injection rate of 0.05 ml/s was used until the entire syringe content was injected. Each viscosity level was tested twice, resulting in eight injections. To make the injections with the two higher viscosities possible, we replaced the 11G cannula with a 16-cm, 8G pipette (Scientific Commodities Inc., Lake Havasu, AZ), as the required pressure was larger.

After the tests, the blocks were scanned again and the resulting CT volumes were registered to those obtained before the tests using intensity-based affine transformations (Analyze, Mayo Clinic, Rochester, MN). The pre-injection CT volume was subtracted from the post-injection CT volume to identify the cement cloud and an isosurface was created from the difference volume to represent the surface of the injected cement. The isosurface was essentially a triangulated mesh that was fit to the surface of the volume composed of the voxels containing cement, and was created using standard functions (MATLAB R2012b, Mathworks, Natick, MA). Also the cement shape in the X-ray images was segmented by subtracting the first image (containing no cement) from the rest of the images in the video sequence.

#### Simulations

**Figure 3:**
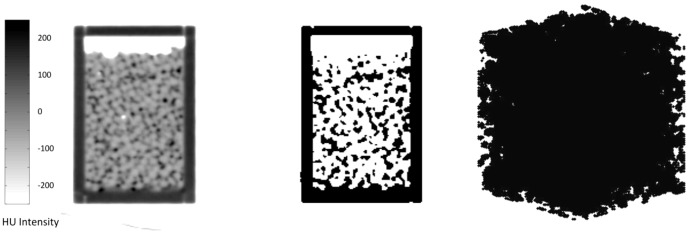
Sample original (A) and masked (B) CT slice and an example of fixed particles arrangement (C).

To simulate the injections, we employed the SPH method proposed in the previous sections. Pre-injection CT scan volumes of the blocks were loaded and a threshold mask was applied to determine the solid regions within each volume. For each scan, any voxel having a Hounsfield Unit (HU) value of greater than zero was assumed to represent a small solid region and a fixed particle was put at the center of that voxel. Assuming the fixed particles occupy the volume of the voxel V, each fixed particle was assigned a mass of ρ_0_V, with ρ_0_ = 1.18 gr/cm^3^ for bone cement [39], the same as the density of fluid particles injected later. Each voxel, based on CT scans acquired, had the dimensions 0.47X0.47X0.5 mm^3^. To reduce the calculations for an unnecessarily large number of fixed particles, a 40X40X40 mm^3^ volume located at the center of each block was taken into account. This volume, however, was large enough to ensure it contained the cement at all times. Figure 3 illustrates a sample arrangement of the fixed particles.

During simulations, fluid particles were added to the scene at the location of the cannula insertion in the corresponding experiments. The rate of adding fluid particles was determined based on their initial volume and the rate of injection. New fluid particles were assigned an initial velocity v_0_ = Q/(πD^2^/4). The states of the fluid particles (total number, positions and densities) were recorded at 1-second intervals, as well as at the end of each simulation. We then used that information to update the CT volumes as if actual CT scans were taken at the time of injections: for each voxel, we found the neighboring fluid particles and used Eq. 2 to determine the fluid density at the center of the voxel. We then increased the voxel HU intensity proportional to the calculated density. A kernel radius of h = 2r was used here, where r is the initial radius of the fluid particles.

Similar to the procedure described before, the pre-injection image volume was subtracted from the simulated one to segment the simulated cement cloud and an isosurface was created. We compared the resulting isosurface with that created from the experimental data as follows [40]: in the isosurface pair, for each point belonging to one isosurface, the closest point belonging to the other isosurface was found and their distance was recorded. For the set of the distances, mean, maximum and standard deviation was calculated. Since the two isosurfaces are likely to contain different numbers of points, this comparison was performed both ways and the larger mean was taken as the ″error″ between the two isosurfaces. We also calculated the mean spreading distance of the cement, similar to the 2D approach of Loeffel et al. [36]: for each voxel containing cement in the CT volume, the distance from its center to the location of injection was found and the mean among all the voxels was calculated. This was repeated for all the simulated and experimental volumes and the experiments were compared with the simulations. To compare the pressure profiles of simulations and experiments, we estimated the SPH pressure at several points in the vicinity of the simulated cannula tip (Eq. 6) and took the average as the injection pressure. Mean pressure in the first 0.5 s of simulation was assumed as the ″zero″ pressure and was subtracted from the subsequent values. This was done to compensate for the erroneous pressure due to the small number of particles in the beginning of the simulations. Also, knowing the viscosity of the cement during the injection as well as the injection rate and cannula geometry, we estimated the pressure encountered by the cement upon exiting the cannula in the actual experiments. Pressure values were then normalized by the maximum value found in the eight experiments. We compared the temporal pressure profiles and the final values between the simulations and the experiments. Finally, we created Digitally Reconstructed Radiograph (DRR) images [41] from the simulated image volumes of the blocks during the SPH simulations. Similar to the X-ray images, we segmented the simulated cement in the DRRs by subtracting the first DRR image from the rest of the images. The resulting images were then compared to the segmented X-ray images.

To determine how fluid particle size affects the accuracy, we performed several of the aforementioned simulations for two sample extreme viscosity levels, changing the initial radius of the fluid particles. We chose values of 1.00, 0.75, 0.57, 0.50 and 0.45 mm for the particles radius and performed the simulations and compared the results with the experimental data as described before. Particle size below which there was no significant improvement in the accuracy was used for the rest of the simulations to avoid unnecessarily costly computations.

## Results

**Figure 4:**
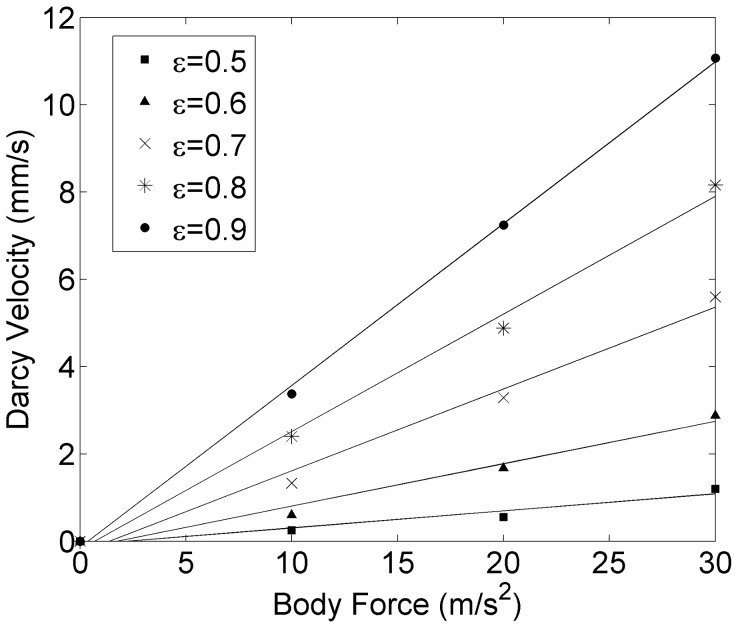
Representative steady-state Darcy velocity as a function of body force for various porosities.

First we show the results of the benchmark simulations performed to compare the performance of the current model and literature data. Figure 4 shows the resulting Darcy velocity for various body forces applied to the fluid. The plot corresponds to the fluid with viscosity of 10 Pa.s and initial particle spacing of 0.5 mm. Other viscosities and particle sizes showed a similar trend. The slope of the best line fit to the points was used to determine ū/F_B_ in Eq. 12 for calculating permeability.

**Figure 5:**
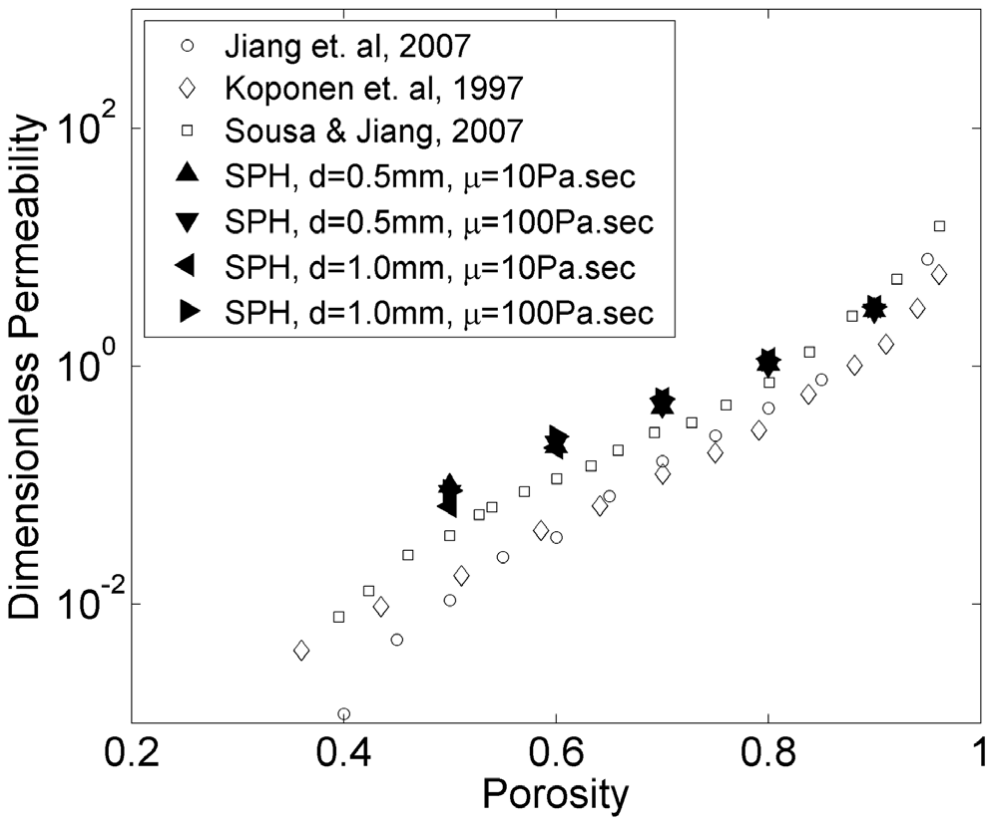
Dimensionless permeability vs. porosity.

In Figure 5 permeability is plotted against porosity, both for the results of the current study and values from the literature [29], [42], [43]. Simulations closely follow the trend of the data from the literature. The agreement is better in the interval of 0.7–0.9 of porosity.

**Figure 6:**
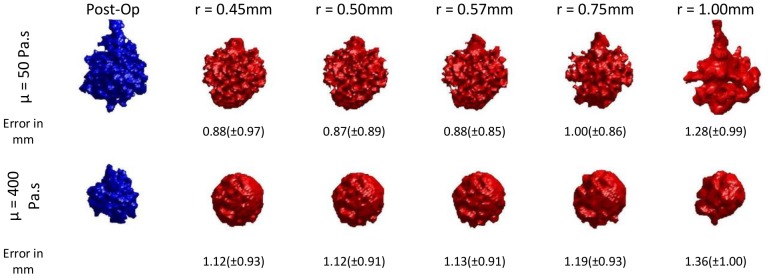
Effect of particle size on the accuracy of the cement cloud.

Figure 6 shows the effect of the particle size on the final cement cloud shape for the foam block tests. The simulated isosurfaces are compared to the isosurfaces extracted from the post-operative CT image volumes acquired from the blocks. Reducing the radius of the particles below 0.57 mm has almost no effect on the accuracy of the resulting cement cloud for either of the viscosity levels. Therefore this particle size was used for the rest of the simulations.

**Figure 7:**
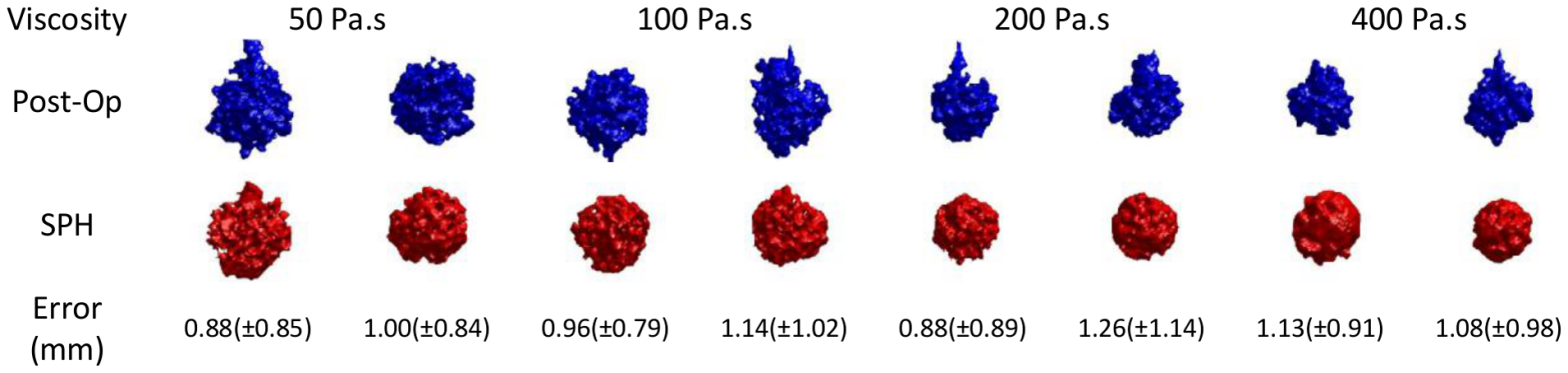
Comparison between isosurfaces of post-operative CTs and simulated image volumes.

Figure 7 compares the simulated and real cement shapes for the eight experiments. Near-millimeter accuracy was achieved in all simulations. The average of all the mean errors is 1.04 mm. There is no evident trend of change in the error as the viscosity increases and this shows the consistency of the simulations for various viscosity levels.

**Figure 8:**
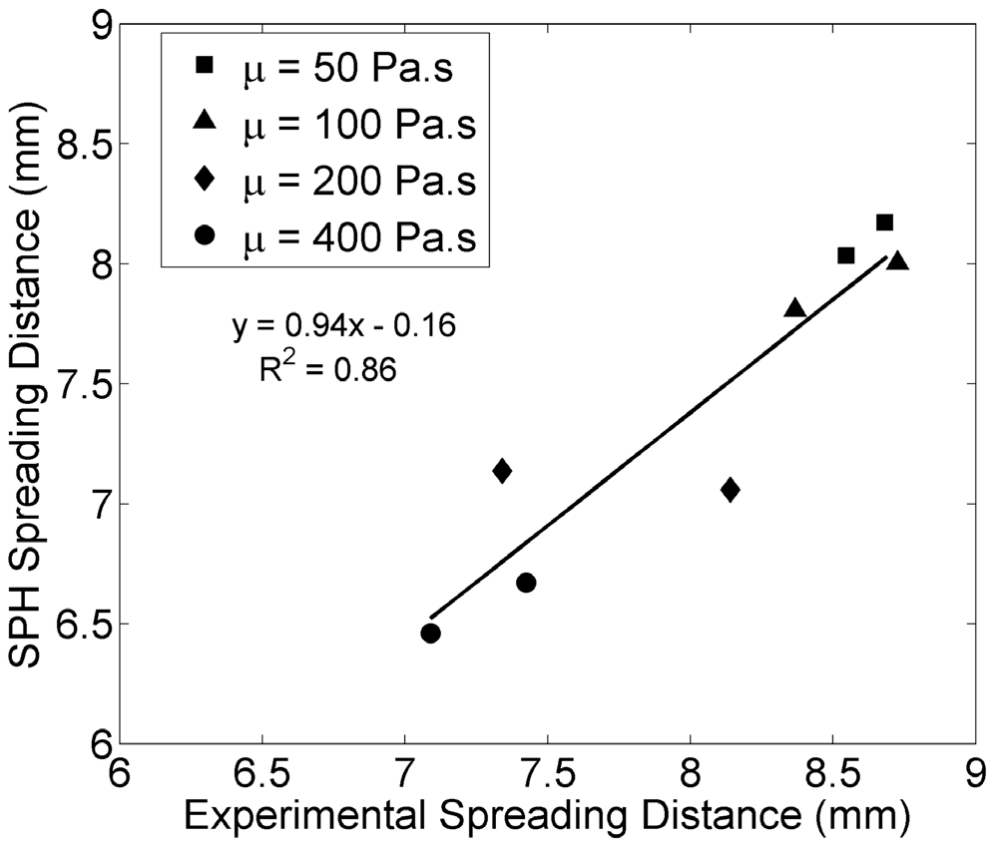
Simulated vs. experimental mean cement spreading distance.

**Figure 9:**
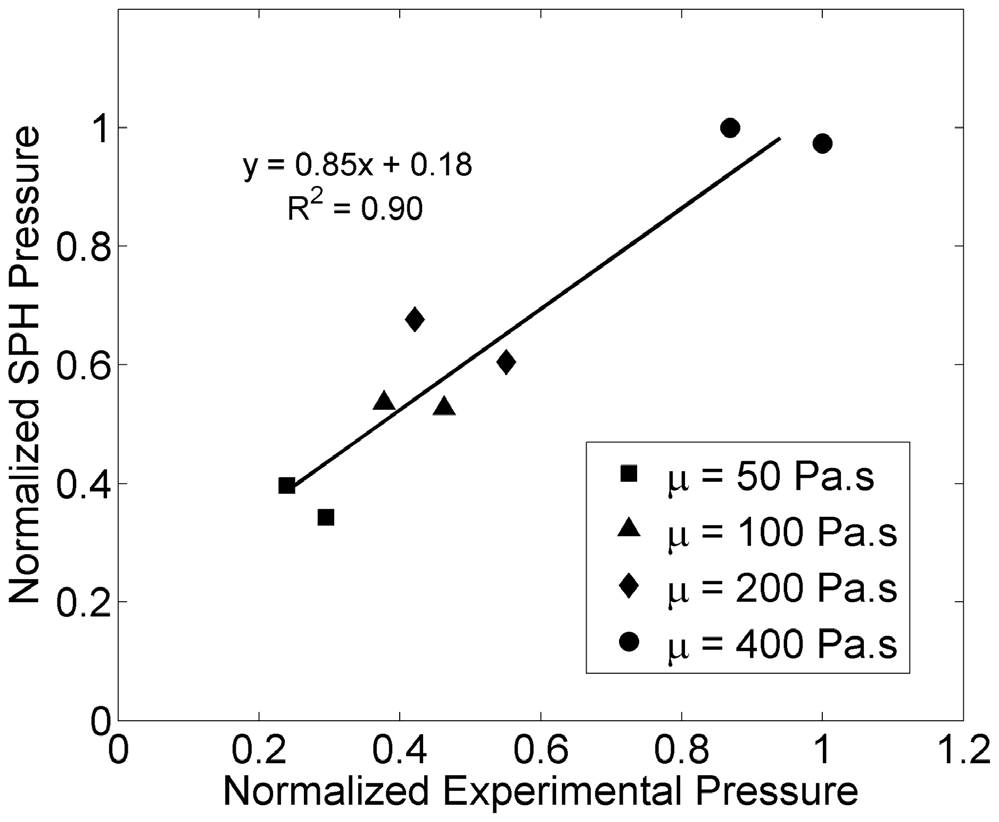
Normalized outlet cement pressure compared between experiments and simulations.

Figure 8 and Figure 9 show comparisons between simulations and experimental values of the mean spreading distance and normalized end pressure, respectively. Strong correlations were found between simulated and experimental values. Spreading distance drops significantly when increasing the viscosity from 100 to 200 Pa.s, while pressure shows a leap when increasing from 200 to 400 Pa.s.

**Figure 10:**
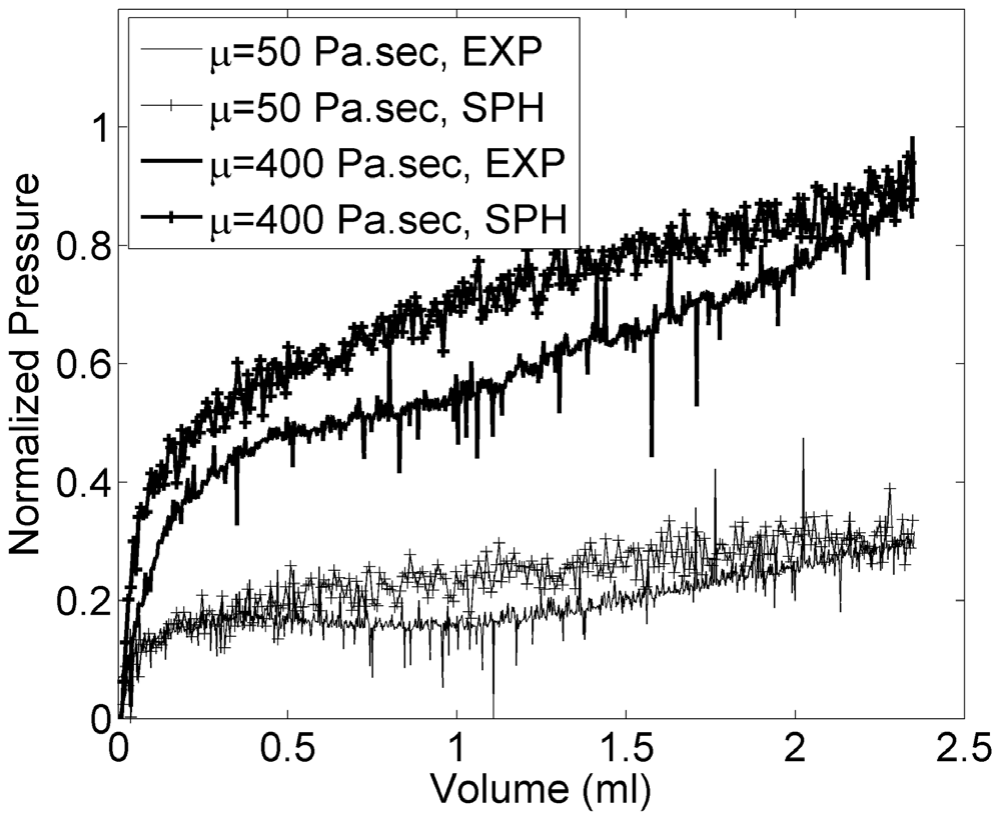
Normalized pressure over the course of injection for example viscosity extremes.

Figure 10 shows example pressure profiles for the two extreme viscosity cases of 50 and 400 Pa.s and compares the trends observed in simulations and experiments. Simulated pressure profiles closely resemble those observed in the experiments. Same trends were seen for the other tests.

**Figure 11:**
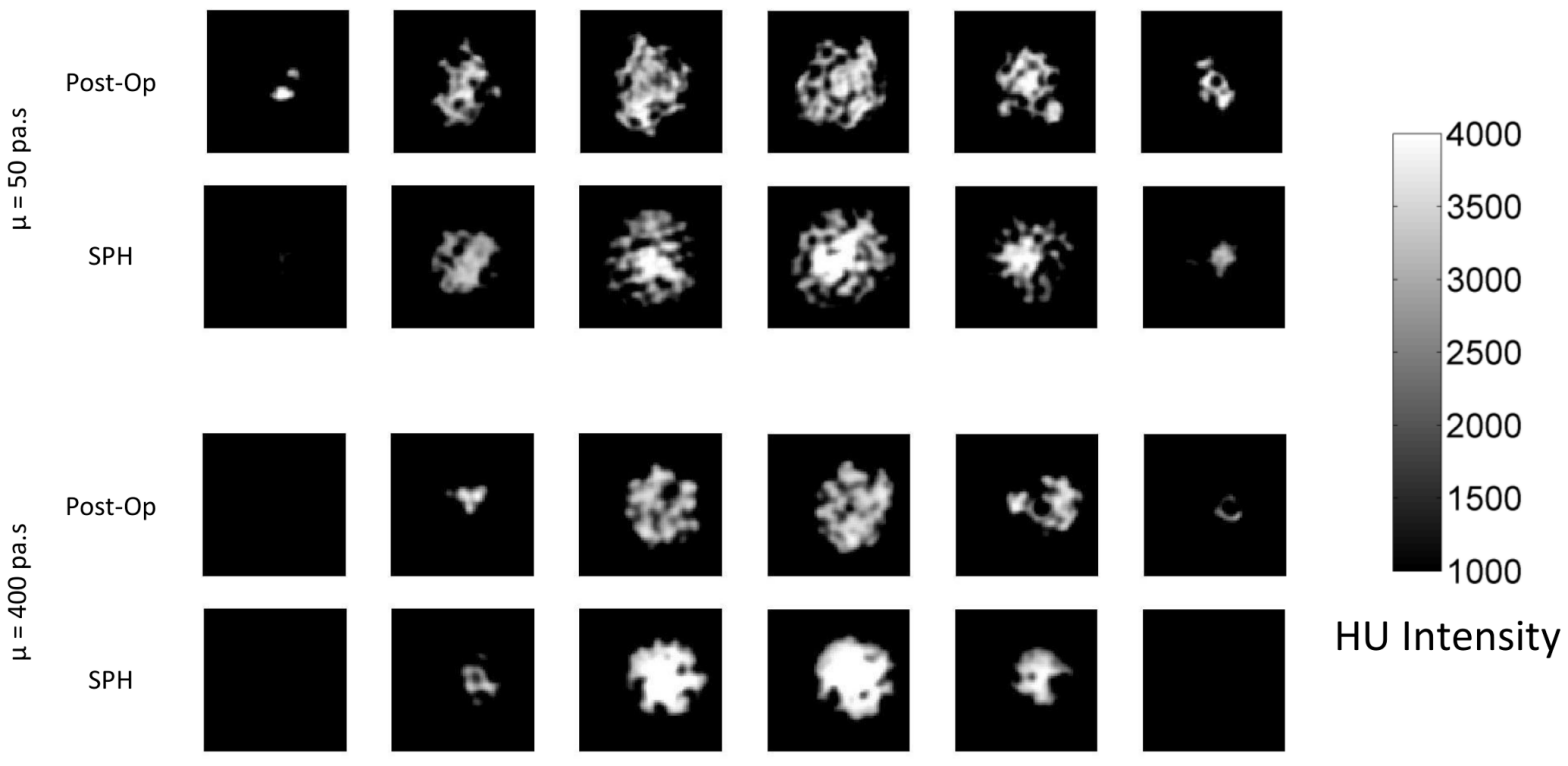
Sample slices taken at 5-mm intervals from the post-operative and simulated CT volumes.

Sample slices of the post-operative and simulated CT volumes for two example injections with extreme viscosities are shown in Figure 11. The figure shows, qualitatively, the different spreading patterns of high and low viscosity cement inside the porous block. Cement with higher viscosity has created a compact shape, while the one with lower viscosity has more articulations and voids when spreading inside the porous foam block. These features were captured closely by the simulations.

**Figure 12:**
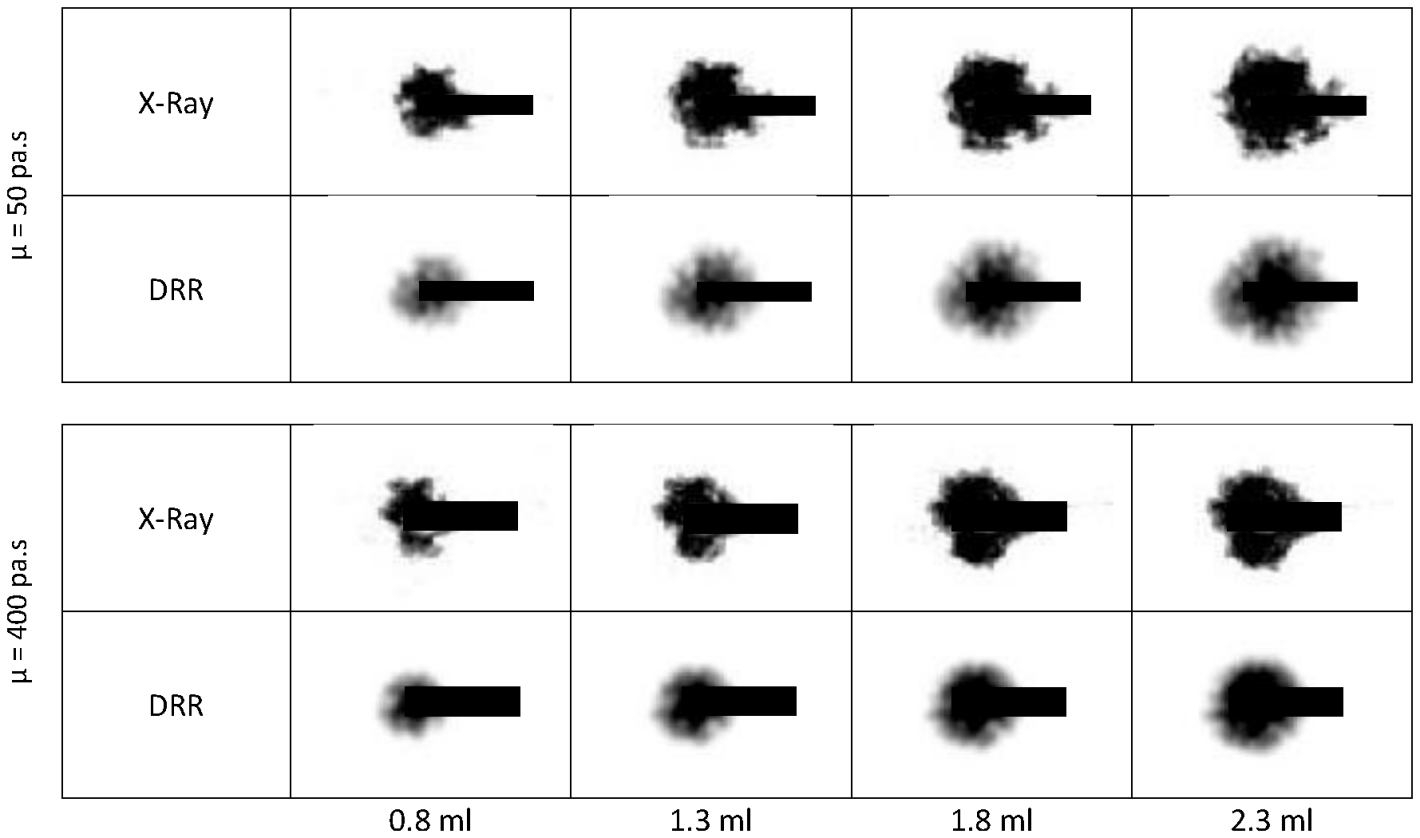
Segmented X-ray snapshots and the corresponding segmented DRR images for two extreme viscosity injections. The dark lines represent the cannulae.

Finally Figure 12 shows snapshots of the X-ray image video sequence captured during injections with the two viscosity extremes and compares those with DRR images created from the corresponding simulated image volumes. Again, one can notice the difference between the two injections as the high viscosity cement creates a denser shape while the low viscosity injection, at equal volumes, tends to spread more and create a less compact shape inside the porous medium. The algorithm for creating the DRR images tends to smear out the edges of the cement shapes, as can be seen in the figure. Nonetheless, the simulations exhibit the same behavior as the real cement in terms of the area of spreading as a function of cement viscosity.

## Discussion

Augmentation of osteoporotic femur using PMMA bone cement to prevent or reduce the risk of fracture has been suggested to be an alternative preventive treatment [2]–[4]. Because of the possible complications, however, the procedure requires precise planning and execution. Effective planning relies, among others, on an accurate method for predicting the diffusion of the cement through the porous medium of osteoporotic cancellous bone. Our goal was to develop a cement diffusion model and validate its efficacy through simulations and experiments. We chose the method of SPH as it has shown potential for modeling of flow of viscous fluids through porous media. Because of the extreme viscosities involved in injecting bone cement, we chose an implicit numerical integration which forced us to modify the usual approximate viscous force calculations typically performed in SPH simulations.

To validate the model, first we performed simplified one-dimensional porous flow simulations and compared the resulting dimensionless permeability values with those obtained in similar studies. Close agreement with the data gathered from the literature was observed. The agreement improved especially in the range of porosity between 70% and 90% which, reportedly, includes the range of cancellous bone porosity [33]. The results were independent of particle size and viscosity which is in agreement with the law of Darcy. However, direct comparison between the simulations and published experimental measurements of bone permeability were not possible for two main reasons: a) there is a wide spectrum of permeability values reported for the cancellous bone in the literature and b) converting those values to dimensionless permeability requires precise knowledge of trabecular structure and distribution for the corresponding experiments.

For the above reasons, we performed an experimental set of validation tests using commercial porous foam blocks as surrogate cancellous bone tissue and injected medical PMMA cement inside those media in a controlled manner. Four viscosity levels were used and the simulations and experiments were compared together. We studied the sensitivity of the results to particles' initial size and chose the largest particle size that provided the best accuracy. Millimeter accuracy was obtained in reproducing the injected cement shape and there were also strong correlations between experiments and simulations for spreading distance and pressure values. Furthermore same temporal pressure profiles were seen in simulations and experiments.

As mentioned in the results section, increasing the viscosity from 100 to 200 Pa.s affected the compactness of the final shape the most, while maximum pressure did not increase by a large factor before increasing the viscosity to 400 Pa.s. This suggests that a viscosity of close to 200 Pa.s is ideal for injections inside porous media including osteoporotic cancellous bone, as the final shape will be compact enough while the required pressure will not be prohibitively large. In the 2D study of Loeffel et al. [36] such large increase in compactness was observed earlier when viscosity changed from 50 to 100 Pa.s. Of note is that their results were averaged over a larger number of experiments with various porosities and injection rates. Also, there, viscosity was estimated with an injection rate of 0.1 ml/s over only 2 seconds, while our tests showed that a plateau in the pressure profile, while estimating the viscosity by pressing out cement, will not be achieved until about 5 seconds after the start of injection, especially for the viscosity levels of 200 and 400 Pa.s. Therefore their reported values for viscosity might be underestimations. Furthermore, we used the same injection rate for both viscosity estimation and injection tests. In this way we eliminated, at the cannula level, the effect of shear rate-dependence of the viscosity [33] which could affect the viscosity measurements. We also note that the spreading distance values we found (7–9 mm) as well as the values reported in the said study (7–10 mm) fall within the same small range despite the difference between the dimensions of the two studies.

Our injection tests were performed using only one injection rate of 0.05 ml/s, as our hardware limitations prevented us from injecting higher viscosity cement with larger injection rates. However, the study of Loeffel et al. [36] showed that changes in injection rate, at least in the interval of 0.05–0.15 ml/s, do not have a significant effect on the spread of the cement.

Several factors could contribute to the discrepancies between simulations and experiments. These include model simplifications such as shear rate-independence of the cement viscosity and the linear change of viscosity over time, while it is well documented that commercial PMMA cement exhibits a nonlinear dependence to both shear rate and time, usually captured using a ″power law″ [33]. The effect of these on accuracy is currently unknown to us. It is worth noting that the proposed model will be used in conjunction with Finite Element Analyses (FEA) to predict the effect of various hypothetical augmentation scenarios on mechanical properties of the bone. The sizes of the elements typically used in FEA are larger than the millimeter accuracy achieved in our simulations [44], [45] and, therefore, FEA accuracy is likely to dominate the overall planning results. This needs to be quantified as part of future studies.

The CT scans were acquired at the highest in-plane resolution possible, which was limited by the size of the blocks and the field of view of the scanning device. We expect that a finer CT resolution would increase the accuracy of the simulation results, noting that the trabecular structure, especially in osteoporotic specimens, is very finely spaced. However, increasing the number of voxels increases the number of fixed particles as well, and that slows down the simulations drastically. For instance, having voxels with half the current edge lengths will require eight times more fixed particles. With the current resolution our simulations yielded reasonable accuracy and we believe the added computational cost of finer resolution CT would not be justified. One must take into account that the proposed model is intended for use in pre-operative planning of bone augmentations and computational efficiency is of crucial importance.

In our simulations we ignored the presence of the bone marrow as a fluid that fills the ″empty spaces″ between the fixed particles and is pushed back by the injected cement. Some pilot simulations proved that taking into account such a fluid will have negligible effect on the end results. This, we hypothesize, is mainly due to the fact that bone marrow viscosity is orders of magnitude smaller than the viscosity of the cement (<0.2 Pa.s for bone marrow [46], [47] vs. 50–400 Pa.s for cement in our study) and the interaction between the two fluid particles are minimal. It must be noted that, even without the marrow fluid, our simulations led to reasonable accuracy, therefore we opted to avoid the added computational load of the second fluid particles, because of the reasons discussed above.

The code written for the simulations was not optimized and was executed in a serial manner. The speed of SPH calculations can be improved by parallelizing the code in an efficient way, exploiting the inherent nature of SPH equations [48].

Results of this study suggest that the chosen method of cement diffusion modeling is an appropriate candidate for our intended application of predicting cement diffusion into porous structure of cancellous bone. This study, to our knowledge, is the first one that quantitatively compares the results, in three dimensions, with those obtained in experiments.
